# In-Gel Isolation and Characterization of Large (and Other) Phages

**DOI:** 10.3390/v12040410

**Published:** 2020-04-07

**Authors:** Philip Serwer, Elena T. Wright

**Affiliations:** Department of Biochemistry and Structural Biology, The University of Texas Health Center, San Antonio, TX 78229-3900, USA; wrighte@uthscsa.edu

**Keywords:** agarose, electron microscopy, genomic DNA sequencing, in-plaque analysis, phage therapy of infectious disease, rate zonal centrifugation in sucrose gradients

## Abstract

We review some aspects of the rapid isolation of, screening for and characterization of jumbo phages, i.e., phages that have dsDNA genomes longer than 200 Kb. The first aspect is that, as plaque-supporting gels become more concentrated, jumbo phage plaques become smaller. Dilute agarose gels are better than conventional agar gels for supporting plaques of both jumbo phages and, prospectively, the even larger (>520 Kb genome), not-yet-isolated mega-phages. Second, dilute agarose gels stimulate propagation of at least some jumbo phages. Third, in-plaque techniques exist for screening for both phage aggregation and high-in-magnitude, negative average electrical surface charge density. The latter is possibly correlated with high phage persistence in blood. Fourth, electron microscopy of a thin section of a phage plaque reveals phage type, size and some phage life cycle information. Fifth, in-gel propagation is an effective preparative technique for at least some jumbo phages. Sixth, centrifugation through sucrose density gradients is a relatively non-destructive jumbo phage purification technique. These basics have ramifications in the development of procedures for (1) use of jumbo phages for phage therapy of infectious disease, (2) exploration of genomic diversity and evolution and (3) obtaining accurate metagenomic analyses.

## 1. Introduction

Historically, neglect was typical of the following questions about gel supported plaques used for isolation, cloning and preparative propagation of phages. Is a bacterial host cell smaller than the spaces within the supporting gel, typically an agar gel, in which bacteria were propagating for plaque formation? What is the effect of changing the radius of the effective gel pore (*P*_E_) on phage plaque formation? These questions achieved increased significance during our work [1,2] with the largest known phage, phage G [3,4,5,6,7]. Phage G did not propagate well either in liquid culture or in the traditional agar gels (typically 0.5–0.7%) used as the top layer, plaque-supporting gel during phage infectivity assays. Possibly, the *P*_E_ of the agar gels was small enough to thermal motion-restrict propagation of phage G. If so, one might replace agar with agarose, gel-forming at higher dilution.

The above questions were accompanied by the more general question of how much information one can obtain about phage particles that are still in a plaque-supporting gel. The more rapidly one can (1) obtain plaques of environmental phages and (2) characterize phage particles in a plaque, the more effective is screening of newly isolated phages for several purposes. These purposes include phage therapy of infectious disease [8,9,10,11] and genomic sequence-analysis of the evolution of both phages and their hosts [12,13,14]. Possibly, phage evolution is linked, via horizontal gene transfer, to eukaryotic evolution [15,16,17,18]. 

Here, we both review and augment data projected to help answer the above questions. We propose that data of this type are critical to efficient isolation of large phages, which include those with genomes longer than 200 Kb (jumbo phages [4,5,6,19]) and even larger phages with genome longer than 520 Kb (mega-phages [20,21]). The importance of these answers is illustrated by the fact that no mega-phage has ever been isolated [20,21], although even larger eukaryotic viruses have been isolated [22]. The existence of mega-phages is surmised from assembly of metagenomic sequences [20,21]. 

## 2. Host Bacteria In-Gel: Values of *P*_E_ for Agarose Gels

### 2.1. Fundamentals

The following data indicate that Gram-negative and Gram-positive phage hosts do not fit in the spaces of the 0.5–0.7% agar gels typically used to form phage plaques. This conclusion is derived, first, from the measurement of *P*_E_ for agarose gels. This measurement begins by determining vs. sphere radius the minimal agarose gel concentration that excludes a sphere during electrophoresis [23]. These *P*_E_ values are then used to anchor *P*_E_ values determined from the sieving (gel-induced retardation) of spheres during gel electrophoresis. The most advanced study of the latter [24] yields the following relationship for underivatized, low-electro-osmosis agarose gels cast in 0.025 M sodium phosphate, pH 7.4, 0.001 M MgCl_2_ at 25 °C: *P*_E_ = 148*A*^−0.87^ nm; *A* is the weight percentage of agarose. 

A 0.7% agarose gel, if characterized by this relationship, has a *P*_E_ of 202 nm, not large enough to admit a bacterial cell, such as the host for phage G. Our phage G host is ~500 nm in radius and 2000–5000 nm in length, as is *Escherichia coli* [25], a representative of the enterobacterial hosts. Our host for phage G has recently been found to be a *Lysinibacillus*, although the host was originally labeled *Bacillus megaterium* (Unpublished data of J.A. Thomas, bacterial size-confirmed in [26]). 

Furthermore, agarose is the least electrically charged sub-fraction of agar; the net charge of agar sub-fractions is negative [27,28]. A detailed study by electron microscopy shows that *P*_E_ decreases as the extent of negatively charged derivatization increases for an agarose gel [29]. This conclusion is supported by the *P*_E_ vs *A* relationship for the most electrically charged commercial agarose, HEEO (Lonza): 98*A*^−0.79^ nm [24]. Thus, at any given gel concentration, agar gels have, on average, smaller internal spaces than agarose gels of the same concentration.

### 2.2. Ramifications

In relation to host cell size, the smallness of the projected agar gel *P*_E_’s leads to the following hypothesis. During formation of a bacteriophage plaque in the typical 0.5–0.7% agar plaque-supporting gels, bacterial host cells push aside the gel-forming fibers while the cells are elongating and dividing. This hypothesis has been found accurate by electron microscopy of thin sections of 0.6% agarose gel-embedded, propagating, uninfected Gram-positive and Gram-negative cells [26]. Furthermore, the gel-breaking pressure exerted by elongating bacterial cells results in counter pressure on cells. This counter pressure is in the range of pressures that cause change in state of the bacterial cytoplasm [26], a change that, in turn, might explain an observed [30] altering of phage T3 infection in-gel. 

A factor in controlling *P*_E_ values is decrease in *P*_E_ as temperature of agarose gelation decreases [31]. Lowering of *P*_E_ by low temperature gelation can be seen via the size of an area of phage propagation-induced bacterial lawn clearing [1]. In this latter study, the different Petri plates were adjacent and in contact with the same surface during plaque formation. The following is a ramification of the above results when one is experiencing erratic plaque formation thought to be the result of large phage size. Two approaches are reasonable to solve this problem. Either raise the temperature of gelation or switch from agar to dilute (0.04–0.2%) agarose. We opted for the latter in the case of phage G and routinely use this approach whenever an objective is isolating or propagating jumbo phages.

Host cell motion-restriction in an agarose gel should decrease as the concentration of the gel decreases. Our definition for formation of a gel is the following. After making a hole, the hole remains indefinitely. We typically make a hole with a 10 μL, glass micropipette. We found that the strongest agarose preparations (Seakem Gold, for example, Lonza [32]) formed a gel at a concentration as low as 0.04%. Gel-forming ability was lost between 0.04% and 0.02%. Electrophoresis of cells of *E. coli* does result in migration through a 0.04% agarose gel when the electrophoresis is done at 0.4 V/cm. Indeed, the cells are found to be fractionated by length; the longer the cell is, the lower the mobility is [33]. Cells are observed either macroscopically by light scattering or microscopically by observing pipetted gel in a phase contrast microscope. During loading and electrophoresis, the 0.04% gel is stabilized by embedding it in a frame of a more concentrated agarose gel [33]. The relatively low field prevents previously observed [34] gel electrophoretic trapping.

## 3. Effect of Plaque-Supporting Gel on Phage Propagation: Work for the Future

### 3.1. Plaque Diameter vs. A

**Figure 1:**
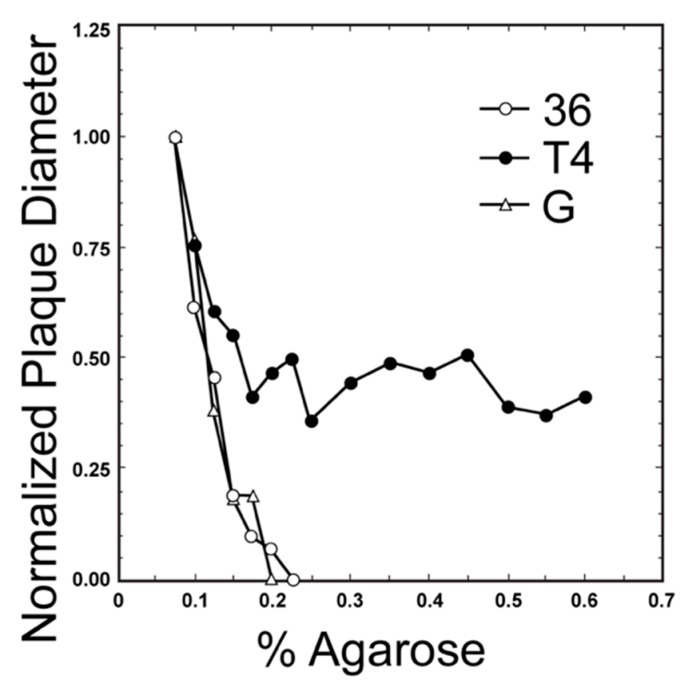
Plots of plaque diameter vs. *A* for phages (adapted from Figure 1 of reference [36]). Key: 36, phage 0305phi8-36; T4, phage T4; G, Phage G.

One can roughly estimate phage size from a plot of plaque diameter vs. supporting agarose gel concentration. Phage size increases as the slope of this plot increases, as seen by comparing the plot for near-jumbo myophage T4 (168.903 Kb genome [35]; Figure 1 [36]) to the plot for myophage G (626 Kb genome [6]; Figure 1) and podophage T3 (38.208 Kb genome [37]); the latter plot is horizontal (not shown in Figure 1). However, when comparing jumbo myophage G to jumbo myophage 0305phi8-36 (218.948 Kb genome [38]; Figure 1 [36]), this relationship is lost in that one visually observes no significant difference in slope, even though (1) the surface area of phage G (cryo-EM data in [6]) is over 2x the surface area of phage 0305phi8-36 (cryo-EM data in [39]) and (2) gel sieving is best correlated with particle surface area [40]. Thus, we conclude that at least one factor beyond sieving has a significant effect on data for jumbo phages. One possible plaque diameter-reducing factor is phage aggregation, known to occur in aging jumbo phage 0305phi8-36 plaques [41].

We offer the hypothesis that another, more biological factor is the length of the tail. Increasing this length would increase sieving. However, increasing tail length would also increase the distance across which a phage particle can reach to infect a cell, which increases in impact as host and phage are increasingly immobilized within a gel. Therefore, increasing tail length could increase plaque size via increasing the probability per time that gel-embedded phages initiate an infection. This is the theoretical source of the above hypothesis. The empirical sources are the plot of Figure 1 and the observation that the 453 nm long phage G tail [4] is about the same length as the 486 nm long [39] phage 0305phi8-36 tail. 

**Figure 2:**
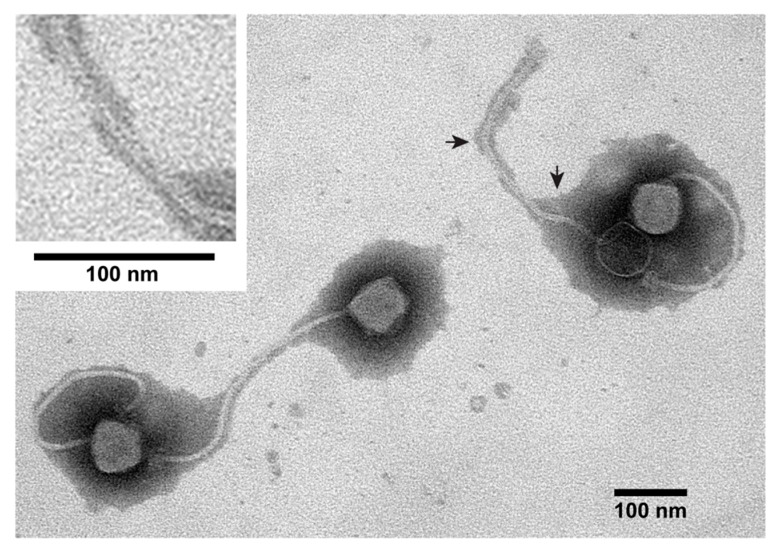
Electron microscopy of *Bacillus pumilis* siphophage 0104phi1-1. The sample was prepared by negative staining with 1.0% uranyl acetate [2]. The inset has a higher magnification image of the section of a tail indicated by the two arrows.

Parenthetically, the hypothesis of the previous paragraph implies that, as environmental phage mobility restriction becomes more extreme and pervasive, tails evolve to become longer and more flexible. Assuming this to be the major reason for long, flexible tails, screening for such tails also screens for biofilm-inhabiting/propagating phages. Figure 2 shows an electron micrograph of *Bacillus pumilus* siphophage 0104phi1-1, which has a longer than usual (~370 nm), more-flexible-than usual tail (other examples: Figure 2 of [42]). This phage is sometimes seen in pairs, as in Figure 2. One of the phages of Figure 2 has injected DNA, which makes capsid interior fill with negative stain and makes the DNA channel in the tail visible, the latter as better seen at higher magnification in the inset for Figure 2. Further work is needed to establish how correlated long, flexible tails are with a biofilm habitat.

### 3.2. Possible Implications

At the risk of over-extrapolating, we note that the above scenario implies that some phages inhabit biofilms without destroying them. Surprisingly, before the recent studies via metagenomics [20,21], one study did perform a test of this aspect. This test was performed by electron microscopy of thin sections. The result was that one sample of human dental plaque had phage-like particles, most of them aggregated [43], that were (1) in concentration that we estimate to be over 10^14^ per ml and (2) jumbo in size. 

Returning to the main theme, we do not have sufficient data to establish a hard principle for using plaque diameter vs. *A* data to determine phage size and type. However, the current data point to the use of these plots as an initial screen for both jumbo phages and typical podophages, such as the related podophages, T3 and T7. The latter are found to be, on average, relatively small. This screen for podophages is not perfect in that small myophages can also pass [44]. However, we know of no example of a jumbo phage or near-jumbo phage that behaves as a smaller, T3/T7-like podophage in a plaque diameter vs. *A* screen. Usefulness of this screen is suggested by the observation that lytic podophages are found dominant in phage cocktails successful in treating of *E. coli* bacteremias [45].

Thus, the possibility exists that a plaque diameter vs. *A* screen will be sufficient to identify jumbo and larger phages and to exclude them when use of smaller phages has a better prognosis. Further work is needed to determine the level of confidence and the extent of size discrimination of screens of this type. The value of a plaque diameter vs. *A* screen, even if imperfect, is increase in the efficiency (in cost and time) with which podophages, jumbo phages and mega-phages are discriminated. Further screens are in Section 5 and Section 6. 

Finally, we note that jumbo phages appear to have been sometimes preferentially selected for phage therapy cocktails, historically. The following is a quote from a manuscript [46] on the phage therapy of typhoid fever. “These two phages, producing very small plaques, possess, however, a remarkable power of diffusion (attack), resulting in plaques of 5 mm. in vitro with homologous cultures.” The last part of this statement is imprecise. However, the first part appears correlate small plaques with phages superior for phage therapy of typhoid fever. Probably, the phages involved were jumbo or near-jumbo phages. The precision of this historical statement would presumably have been higher if the association of small plaques with large phages had been known.

## 4. Other Advantages of Using Dilute Agarose Gels for Initial Phage Propagation

As previously mentioned [39], phage isolation/propagation exclusively in dilute agarose gels has additional advantages. These advantages are derived, in part, from isolation procedure that includes (1) flooding a dry environmental sample with a molten agarose-host cell mixture (no filtration, centrifugation or chloroform treatment), (2) gelling this mixture and (3) incubating until clear regions appear within the lawn formed by the bacteria (detailed illustration: [47]). The host cells suppress most growth of bacteria from the environmental sample [1]. The advantages include isolation of phages that (1) require high local concentration of host cells to elute phages from environmental particles to which the phages have bound, (2) are out-propagated by other phages in liquid culture and (3) have propagation stimulated by dilute polymer, such as agarose. This procedure will be called “phage isolation via gel-embedded sample”. The need for procedure with these advantages has been discussed [9]. 

Furthermore, the elimination of chloroform treatment, filtration and centrifugal pelleting removes bias against isolation of phages removed or inactivated by these procedures. This point is illustrated by the dramatic bias against membrane coated phages introduced by the use of chloroform [48]. Use of filtration is an especially bad practice when isolating jumbo phages.

We note that, as originally isolated, *Pseudomonas chlororaphis* (jumbo) phage 201phi2-1 is one of the phages with propagation stimulated by dilute agarose gels. This phage initially propagates actively in liquid culture. However, before a liquid culture visibly loses turbidity, 201phi2-1 stops propagating [44]. This phage could not have been isolated by either conventional liquid enrichment culture or single-plaque procedures with 0.5–0.7% agar upper layer gels. It has the longest genome (316.674 Kb [49]) of any of the 15 *Pseudomonas* jumbo phages [50]. 

Furthermore, selective pressure to lose or alter laboratory-non-essential 201phi2-1 genes was minimized by minimizing serial in-laboratory propagation [44]. This practice is expected to assist retaining of activity of gene products not essential in the laboratory. This retention is an advantage for both basic science (e.g., [51], if any involved genes are non-essential) and phage therapy. Minimizing serial laboratory propagation should, in our opinion, be routine practice.

Parenthetically, we assume that, similarly, if one serially laboratory propagates a phage host, the host genome will be altered. For example, when we tried phage isolation via gel-embedded sample with our laboratory *Escherichia coli* BB/1 strain, bacteria endogenous to soil samples were not adequately suppressed. This *E. coli* strain has presumably become weakened by laboratory propagation.

As with studies of plaque diameter vs. *A* plots, studies of polymer-stimulation of phage propagation are far from complete. Only one polymer, agarose, has been tested and only with three hosts. The cause of stimulation is not known. One hypothesis is that hydrated polymer is a signal to phages of the presence of a hydrated host. Given that the exposure of phages to hydrated polymers occurs in biofilms [52,53], the data suggest a biofilm habitat for phages with propagation stimulated by hydrated polymer. 

## 5. Further Screening In-Plaque: Native Gel Electrophoresis (AGE) and Fluorescence Microscopy

Especially for phage therapy of bacteremias, high persistence in blood is, in theory, a usefulness-promoting phage characteristic. The data indicate that murine blood persistence varies by orders of magnitude among different phages. Phage T3 has the highest known persistence with detectable blood titer loss not occurring for 3–4 h [54]. Thus, rapid screening for high persistence is possibly going to become rate limiting for the speed at which phage therapy cocktails are assembled. However, direct measuring of persistence is, itself, so time-consuming that a more rapid, proxy screen is needed. 

Here, again, we do not have enough data to provide a complete picture of how to proceed. However, if one considers the limited data/ideas that we do have, a direction is suggested. Specifically, most (not all) blood vessel linings have a negative average electrical surface charge density, σ [55]. Thus, a logical proposal is that, as the magnitude of the negative σ of a phage increases at physiological pH, the binding of the phage to at least blood vessels will decrease. Predicting phage loss via cellular uptake is a more complex enterprise [56]. More comprehensive comparison of persistence to σ is needed to determine the effectiveness of a σ screen for high phage persistence. 

Fluorescence microscopy of DNA-stained particles in a phage plaque is efficiently (in time and cost) done and reveals additional information. First, phage aggregation within a plaque differs dramatically among phages, as (easily) seen by in-plaque fluorescence microscopy of DNA-stained particles [41]. Unfortunately, we do not have direct information about the linkage between in-plaque phage aggregation and other phage characteristics, such as history of biofilm existence. In the above-cited study of the thin sections of human dental biofilm, the phages were mostly aggregated. Additional studies of this type are needed to determine how widespread is the association of phage aggregation with biofilm habitation.

Finally, in-plaque fluorescence microscopy can be used to identify jumbo phages and mega-phages via (1) in-plaque thermal motion of phage particles (The larger the phage is, the smaller its thermal motion), (2) the brightness of DNA-stained phage particles and (3) the length of DNA accidentally expelled from capsids and observed by fluorescence microscopy after stretching DNA molecules by pressing on a cover glass. This latter procedure is remarkably simple to use if one has a fluorescence microscope [57]. 

## 6. Screening In-Plaque: Electron Microscopy

Electron microscopy (EM) of thin sections (EM-TS) of phage plaques can classify and size-characterize phages, while it also informs about the phage life cycle. EM-TS is especially useful when local access to technology is limited. The reason is that, after obtaining phage plaques, local resources are needed only for plastic-embedding (typically Epon-embedding) of an excised piece of a plaque-supporting gel. The embedded, plaque-supporting gel can then be shipped, without hazard to the sample, to a center that has the facilities for the TS, EM and the interpretation of images.

**Figure 3:**
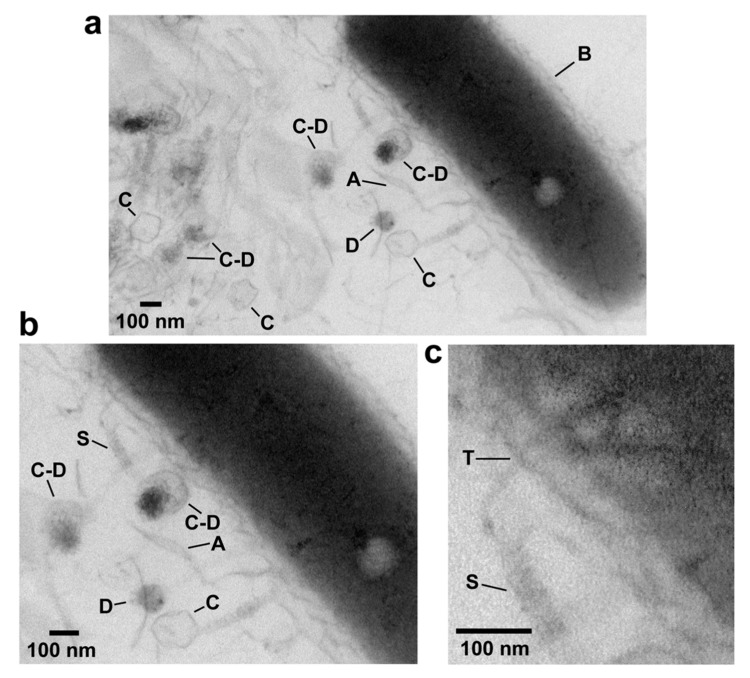
EM-TS of a phage G plaque. The procedure of reference 28 was used to embed, thin section and perform electron microcopy of a plaque of phage G. (**a**) A field at relatively low magnification, (**b**) a region within the same field at higher magnification, (**c**) a region of the field of (**b**) at higher magnification and with increase in contrast. A, agarose fiber; C, capsid; D, condensed DNA; S, tail sheath; T, tail tube; B, bacterial cell.

In-plaque EM-TS is effective for non-tailed dsDNA phages [48] and podophage T3 [30]. Figure 3 shows effectiveness for myophage G. Several G capsids (C in Figure 3a), some associated with condensed DNA (C–D) are in this image. In addition, a bacterial cell (B in Figure 3a) is present. Agarose fibers are also visible, especially at higher magnification in Figure 3b (A in Figure 3a,b). The DNA molecules are condensed in a spherical conformation (D in Figure 3a,b). The DNA sometimes appears to have separated from an associated capsid while remaining condensed (lowest C and D in Figure 3a,b), for reasons not known. 

The phage is identified as a myovirus by a long, contractile tail sheath on some C particles. The sheath is most easily seen at even higher magnification (S in Figure 3c). A tail is not seen on some C particles in Figure 3a. The tail might be present, but not adequately in the plane of the section. The phage is identified as jumbo by the diameter of its capsid, 139-152 nm. Observed diameter has been shown for other phages to reflect ~12% shrinkage that occurs during dehydration for Epon embedding before thin sectioning [58]. 

The following novel detail is in Figure 3. One contracted sheath (S in Figure 3b,c), surrounds an inner tube (T) that extends into the central region of cytoplasm of an attached cell, as best seen in the magnified and contrast-enhanced image of Figure 3c. This tube is presumably a conduit for DNA being injected into the bacterial cell. 

## 7. Preparative In-Plaque Propagation of Jumbo Phages

For purifying jumbo phages G, 201phi2-1 and 0305phi8-26 in amounts sufficient for chemical and physical characterization, in-plaque propagation is our method of choice. The procedure for preparative in-gel propagation is the procedure for plaque formation, except that the number of plaques is made higher. If plaques are uniformly distributed, the optimal number is typically over 1000 and depends on the plaque size and, therefore, on the concentration of the supporting gel. Alternatively, a relatively rapid, simple means for seeding phages for preparative propagation is to (1) phage-load a platinum needle by stabbing a plaque, then, (2) stab, 2-4 times, the phage-loaded needle into Petri plate-contained bottom agar, at a single location, and (3) repeat at 6–9 locations. Pour a mixture of growth medium, host bacteria and molten agarose across the phage-inoculated bottom agar. Finally, spread the molten agarose mixture with one gentle rocking motion and allow to gel. Relatively large plastic Petri plates, 15 cm in diameter, can be used to increase phage amounts. 

After gelation of the dilute agarose overlay, incubate the Petri plate to produce the in-gel phage lysate. Incubation is continued until lysis is almost confluent, with bacterial turbidity seen only at the perimeter of the upper layer gel. Harvest the agarose gel-contained phages. Remove agarose and bacterial cells by pelleting; extract phages from the pellet; repeat pelleting; pool the phage-containing supernatants. Details for this and the previous paragraph have been previously described for phage G [1] and 201phi2-1 [44]. These details will vary for other phage/host combinations. However, we have no indication that a fundamental change in procedure will be needed when host and/or phage are changed. 

In addition to efficiency (of time and cost), reasons for adopting an in-gel preparative procedure of this general type include elimination of liquid culture-associated aerosol formation. Containment procedures are the same as they are for incubating Petri plates used for plaque counts. 

## 8. Purifying Jumbo Phages and Their DNA 

Phage G loses activity (expels its genome) and phage 0305phi8-36 also loses activity (contracts its tail) when attempts are made to purify these jumbo phages by buoyant density centrifugation in a cesium chloride density gradient (unpublished data from our laboratory). Nonetheless, with many other phages, this phage purification procedure is effective and is used. To our knowledge, no systematic study has been made of which phages experience negative effects from centrifugation in cesium chloride density gradients. Given the negative effects already known for some phages, a back-up procedure is needed. 

**Figure 4:**
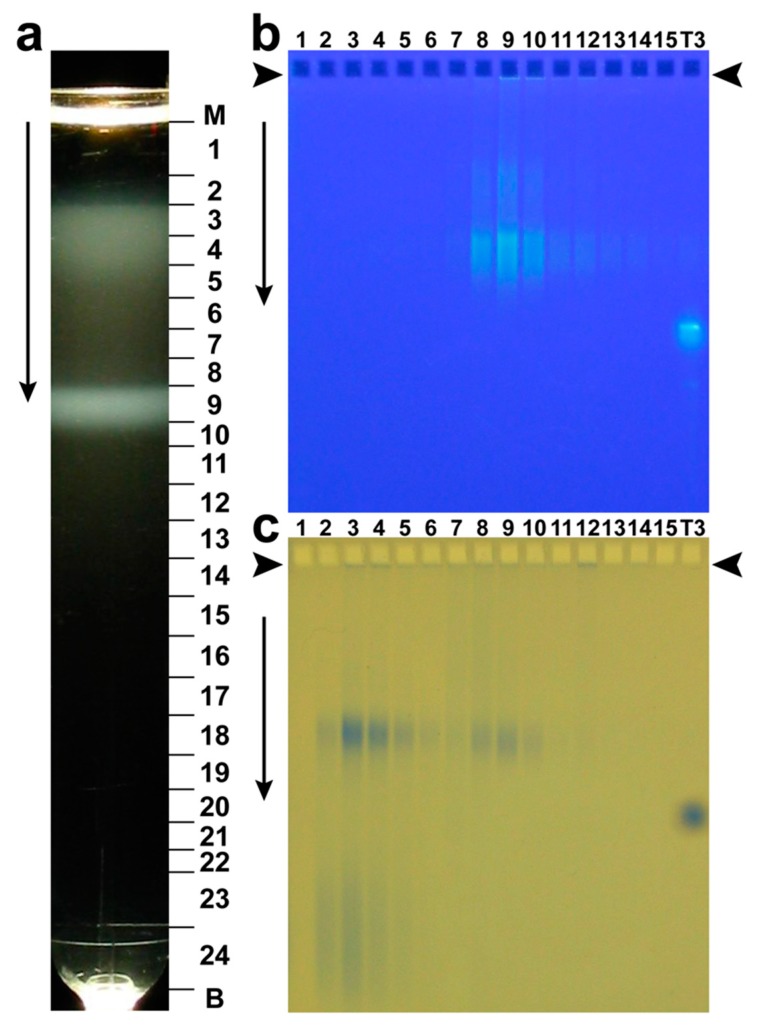
Purification of phage G by rate zonal centrifugation in a 10–35% linear sucrose gradient in the following buffer: 0.01 M Tris-Cl, pH7.4, 0.01 M MgCl_2_, 6% polyethylene glycol 3350 (Beckman SW41 rotor, 14,500 rpm, 1.0 h, 5 °C). The gradient had a volume of 10.8 ml and was poured over a 0.7 ml 62% sucrose shelf. (**a**) Photograph of the light scattering from the centrifuge tube (M, meniscus; B, tube bottom). (**b**) AGE of the fractions of panel (**a**), as indicated at the top of a lane, followed by nucleic acid-specific staining with GelStar. (**c**) The gel of (**b**) after protein-specific staining with Coomassie blue [39,47]. Electrophoresis was performed at 1.0 V/cm for 16.0 h. in a 0.4% submerged agarose gel (Seakem LE) in 0.09 Tris-Acetate, pH 8.3, 0.001 M MgCl_2_. The T3 lane in Figure 4b,c has phage T3 used as a control for band integrity. Some T3 DNA has leaked out of T3 phages; this DNA co-migrates with phage particles during AGE, as seen in the (**b**) panel. The vertical arrows indicate the direction of electrophoresis; arrowheads indicate the origins of electrophoresis.

Historically, phage G was originally purified by rate zonal centrifugation in a sucrose gradient [59]. A variant of this procedure was also non-toxic to phage 0305phi8-36 and was effective [60]. Presumably, other jumbo phages will also be relatively unharmed during purification in a sucrose gradient. An image of the light scattering from phage G after centrifugation through a 10–35% linear sucrose gradient is in Figure 4a. The major band, centered in fraction 9, is formed by phage particles. Two conventionally sized Petri plates were used for preparative phage G propagation.

Confirmation that phages form this band is initially from infectivity titer of the various fractions of Figure 4a (not shown). A technique for physically identifying both phages and other particles is AGE of the various fractions of the sucrose gradient. Figure 4b shows AGE of fractions of Figure 4a, with nucleic acid-specific GelStar-staining. The fraction number from Figure 4a is indicated above a lane in Figure 4b. Closely spaced bands of GelStar-staining (DNA-containing) particles in Figure 4b are in the profiles of fractions 8-10 and are, therefore, formed by phage particles. The formation of more than one AGE band indicates phage particle heterogeneity. The source of the heterogeneity is not known. The phage protein is seen in fractions 8-10 after subsequent staining of the same gel with Coomassie blue (Figure 4c). 

Light scattering was also seen in fractions 3–5 of Figure 4a, closer to the origin of the sucrose gradient. Figure 4c reveals that most protein of these fractions is in particles that co-migrate with phage particles during AGE. These particles do not have nucleic acid, based on non-staining with GelStar in Figure 4b. Electron microscopy of negatively stained specimens (not shown) confirms that these particles are empty capsid shells. 

Finally, we make two related points. First, if genomic sequencing and other DNA analyses are the only objectives, purifying a newly isolated phage is not necessary. Phage DNA can be obtained, without significant host DNA, from a lysate by (1) bursting host cells, without bursting phage particles, followed by (2) digesting the host DNA (The phage DNA is protected by the capsid) and, finally, phenol extraction of the phage DNA. Details are in reference [61]. In our experience, the only complication is the sometimes presence of agarose in the DNA preparation. Jumbo phage DNA is separated from agarose by winding the DNA onto a glass rod, as done by Avery et al. [62]. We do not know whether sequencing can be done in the presence of contaminating agarose. If it can, obtaining sequencing-quality phage DNA is automatable (no winding of DNA on a glass rod) by use of current laboratory robotic technology. This is not now possible if ultracentrifugation is used. 

Second, at least some metagenomic analyses would be more accurate if they used a procedure like the one in the previous paragraph to obtain a metagenomic phage DNA fraction. For example, evidence for the existence of mega-phages does not include any evidence for the presence of a capsid [20,21]. A metagenomic version of the procedure of the previous paragraph would produce that information. If used in general for metagenomic studies, this procedure would reduce distortion caused by the loss of jumbo phages, mega-phages and, presumably, also the membrane-covered phages that are deficient in metagenomic analyses (Section 4). 

## 9. The Significance of Drying Phages

We anticipate that, in the future, improvements in phage storage will assist use of jumbo phages. Phage G, for example, does not store well when frozen. We have had –70 °C-stored phage G preparations lose all activity in 15–20 years. In contrast, G-containing fractions of sucrose gradients remain active for over 20 years at 4 °C, with ~1 log loss of titer. Furthermore, maintaining frozen stocks is expensive. If phages must be shipped cold, logistical problems occur, especially for phage therapy in remote locations. 

A better approach would be to store phages in a dry state. We note that all phages with our nomenclature (month/year/phi/host strain number/phage number in the month indicated) were, thus far, isolated from dry samples obtained in southern Texas, sometimes in the summer, when soil temperatures reached 50–60 °C. Thus, we think it likely that procedure for dry storage is possible, especially for phages from dry environmental samples.

## 10. Conclusions/Perspectives

Although, as discussed above, the jumbo phage isolation story is far from complete, enough has already been done to develop a concept of how one can accomplish increased effectiveness in the isolation, characterization and use of jumbo phages and even mega-phages. This concept begins with dilute agarose in-gel isolation and propagation. At this point, no superior alternative is visible to us. Specifically, all mega-phage isolation attempts have failed and these appear to have used, minimally, 0.25% agar gels ([20,21] and included references). Much larger pores are present in 0.04–0.20% agarose gels. Dilute agarose gels apparently were not used in attempts to isolate mega-phages. Even bacterial cells fit in the internal spaces of a 0.04% agarose gel [33]. Perhaps, the importance of plaque-supporting gel *P*_E_ has been obscured by the fact that bacteria propagate within agar gels as concentrated as at least 1%. However, we now know that the bacteria push gel fibers aside even when propagating in 0.6% agarose gels [30]. Phages cannot do this.

The concept continues with rapid characterization and screening of the phages isolated. Here, we have discussed characterization that is performed with phages still in a plaque. Large phages are most rapidly identified via (1) steep plaque diameter vs. *A* plot and (2) in-plaque fluorescence microscopy. Further rapid characterization is performed via in-plaque EM-TS and genomic analysis. The latter includes both pulsed field gel electrophoresis of the entire phage genome and whole phage genome sequencing. 

For phage therapy of at least bacteremias, probably the highest impact, screening-driven objective is increasing the efficiency of finding phages with high persistence, whether or not phages are jumbo. The AGE-based procedure suggested here needs testing. The objective is to develop screens for either phage therapy potency or capacity to fill gaps in analysis of gene function and evolution. Whole genome sequencing/informatic analysis should eventually become a high-speed procedure. Speed and automation are promoted by phage-specific DNA isolation without phage purification [61].

The above characterizations might yield screens for phages most effective for the therapy of biofilm-carried infections. For example, the untested possibility exists that jumbo phages acquired their large genomes during selection for survival in multiple complex environments, primarily biofilms. Perhaps, therefore, jumbo phages and compounds that stimulate their propagation (such as dilute polymers) should be favored for inclusion in phage therapy cocktails used for biofilm-carried infections. 

Jumbo phage isolations are also needed to answer questions of phage evolution, such as the following. Did phages begin as cells that have subsequently undergone reductive genomic evolution [63,64] or are jumbo phages the products of accretive evolution [6,65] (or both)? In addition, do phages have genes that promote phage evolution to provide fitness of the ecosystem of which the phage is a part [18]? These questions can be answered by comparing related phages that represent various stages of evolution. The idea is to work to establish an evolutionary pathway, in analogy with work to establish phage assembly pathways. Finding of ancient imprints among today’s propagating bacteriolytic particles would be a major assist.

We note that, for phage therapy, the various screens do not have to be perfect. In modern-day practice, phage therapy is already effective with screening only for lytic character [66,67,68]. In any case, phage therapy is done with cocktails of several phages that are not likely to all have the same disadvantages. Similarly, genomic evolution has been studied with minimal pre-sequencing screens. We propose the idea that the next stage of improvements in both areas will be based on rapid, high-throughput screening for optimal phages.
